# Association of the triglyceride-glucose index with dynamic bidirectional transitions of cardiovascular-kidney-metabolic syndrome: a multistate Markov model study based on CHARLS

**DOI:** 10.3389/fmed.2026.1912039

**Published:** 2026-09-28

**Authors:** Xiunan Liu, Buyuan Tian, Hongwei Leng, Xumao Deng, Yun Sun, Xiang Xiao

**Affiliations:** 1 School of Clinical Medicine, Chengdu Medical College, Chengdu, China; 2 Department of Nephrology, The First Affiliated Hospital of Chengdu Medical College, Chengdu, China

**Keywords:** cardiovascular-kidney-metabolic syndrome, CKM progression, middle-aged and older adults, multistate Markov model, triglyceride-glucose index

## Abstract

**Objective:**

To characterize dynamic transitions across cardiovascular-kidney-metabolic (CKM) stages and examine the associations of the TyG index with bidirectional transitions between low- and high-risk CKM states among middle-aged and older adults.

**Methods:**

Data were obtained from CHARLS. A continuous-time multistate Markov model was used to estimate transition intensities, predicted transition probabilities, and expected sojourn times across CKM stages 0−4. A two-state Markov model was further constructed by defining CKM stages 0−2 as the low-risk state and stages 3–4 as the high-risk state to evaluate the associations of the TyG index with CKM progression and reversal.

**Results:**

A total of 5,135 participants were included. CKM stages 0–3 exhibited clear bidirectional transitions, with more active transitions at earlier stages and longer expected sojourn times at higher stages. The predicted 4-year probabilities of transition to CKM stage 4 among participants initially in stages 0, 1, 2, and 3 were 8.3%, 8.8%, 12.0%, and 13.9%, respectively. The expected sojourn time increased from 1.97 years in stage 0 to 10.64 years in stage 3. In the multivariable-adjusted two-state model, compared with the lowest TyG tertile, the HRs for progression from the low-risk to the high-risk CKM state were 1.15 (95% CI: 0.96–1.37) for T2 and 1.35 (95% CI: 1.11–1.65) for T3. Restricted cubic spline analysis showed a significant linear positive association between the TyG index and CKM progression risk. No significant association was observed between the TyG index and reversal from the high-risk to the low-risk CKM state.

**Conclusion:**

CKM exhibited a clear dynamic evolutionary pattern, with greater transition activity at earlier stages and increasing persistence at more advanced stages. Higher TyG levels were associated with progression from the low-risk to the high-risk CKM state but not with CKM reversal. The TyG index may provide complementary information for identifying individuals at increased risk of CKM progression.

## 1 Introduction

In 2023, the American Heart Association (AHA) formally proposed the integrated concept of CKM, emphasizing the complex associations among the heart, kidney, and metabolic systems (1). CKM is not a single disease, but a continuous disease process formed by the interweaving of metabolic abnormalities, kidney damage, and cardiovascular disease. The AHA further developed a staging model from stage 0 to stage 4 to describe the evolution of individuals from having no risk factors, to the accumulation of metabolic risk, and then to subclinical and clinical cardiovascular disease. Epidemiological evidence shows that the burden of CKM is high among middle-aged and older adults in China. Approximately 80% of individuals meet the diagnostic criteria for CKM syndrome, and 23.6% are already in CKM stages 3–4 (2). Therefore, identifying high-risk individuals in the early stages of CKM and delaying their progression to higher stages are important issues in current CKM prevention and control.

In recent years, insulin resistance, as an important pathological mechanism linking metabolic abnormalities and cardiovascular disease (3), has received increasing attention regarding its relationship with the occurrence and development of CKM. The TyG index is a simple indicator calculated from fasting triglycerides and fasting plasma glucose and has been widely used to evaluate insulin resistance (4). Compared with traditional metabolic indicators, the TyG index is easy to obtain, has good reproducibility, and shows stable predictive ability. Previous studies have shown that the TyG index is closely associated with the occurrence of metabolic syndrome (5), type 2 diabetes (6), and atherosclerotic cardiovascular disease (7). However, existing studies have mainly focused on the relationship between TyG and single disease outcomes, with less attention paid to its association with the continuous transition process across CKM stages. In particular, given that CKM has coexisting characteristics of “progression” and “reversal,” longitudinal evidence is still lacking on whether TyG is mainly related to disease progression and whether it is associated with reversal from a high-risk state to a low-risk state.

In addition, traditional epidemiological studies mostly use single-endpoint or static analytical methods, which makes it difficult to fully reflect the dynamic changes in CKM stages during follow-up. Multistate Markov models can handle multiple health states and their transition processes within a unified framework, characterize the dynamic process of disease progression from a low-risk stage to a high-risk stage and reversal from a high-risk stage to a low-risk stage, and quantify the transition intensities between different states. Therefore, this method provides a more refined analytical tool for studying the continuous evolution of CKM.

Based on this, the present study used CHARLS data (8) and applied a continuous-time multistate Markov model to systematically describe the bidirectional transition characteristics of CKM stages under natural conditions. Transition intensities and expected sojourn times between different states were estimated to clarify the natural evolution pattern of CKM syndrome. On this basis, a simplified two-state Markov model was constructed, and the TyG index was further incorporated into the model to quantify its association with bidirectional CKM transitions. This study aimed to reveal the association between the TyG index and the dynamic evolution of CKM, providing a basis for early risk stratification and intervention strategies.

## 2 Materials and methods

### 2.1 Study design and population

CHARLS is a nationally representative longitudinal cohort study conducted by the National School of Development at Peking University. It focuses on Chinese adults aged 45 years and older and aims to investigate population ageing in China and provide evidence for policy development. To date, CHARLS has completed the baseline survey in 2011 and four follow-up surveys in 2013, 2015, 2018, and 2020 (8). This study was approved by the Biomedical Ethics Review Committee of Peking University (approval number: IRB00001052−11015), and all participants provided written informed consent before participation.

A total of 17,705 participants provided blood samples at baseline. The exclusion criteria were as follows:

(1) age <45 years;
(2) missing or abnormal key biochemical data;
(3) missing basic demographic information or anthropometric measurements; and
(4) incomplete follow-up data or loss to follow-up. The final analytical cohort included eligible participants (Figure 1).

**Figure 1:**
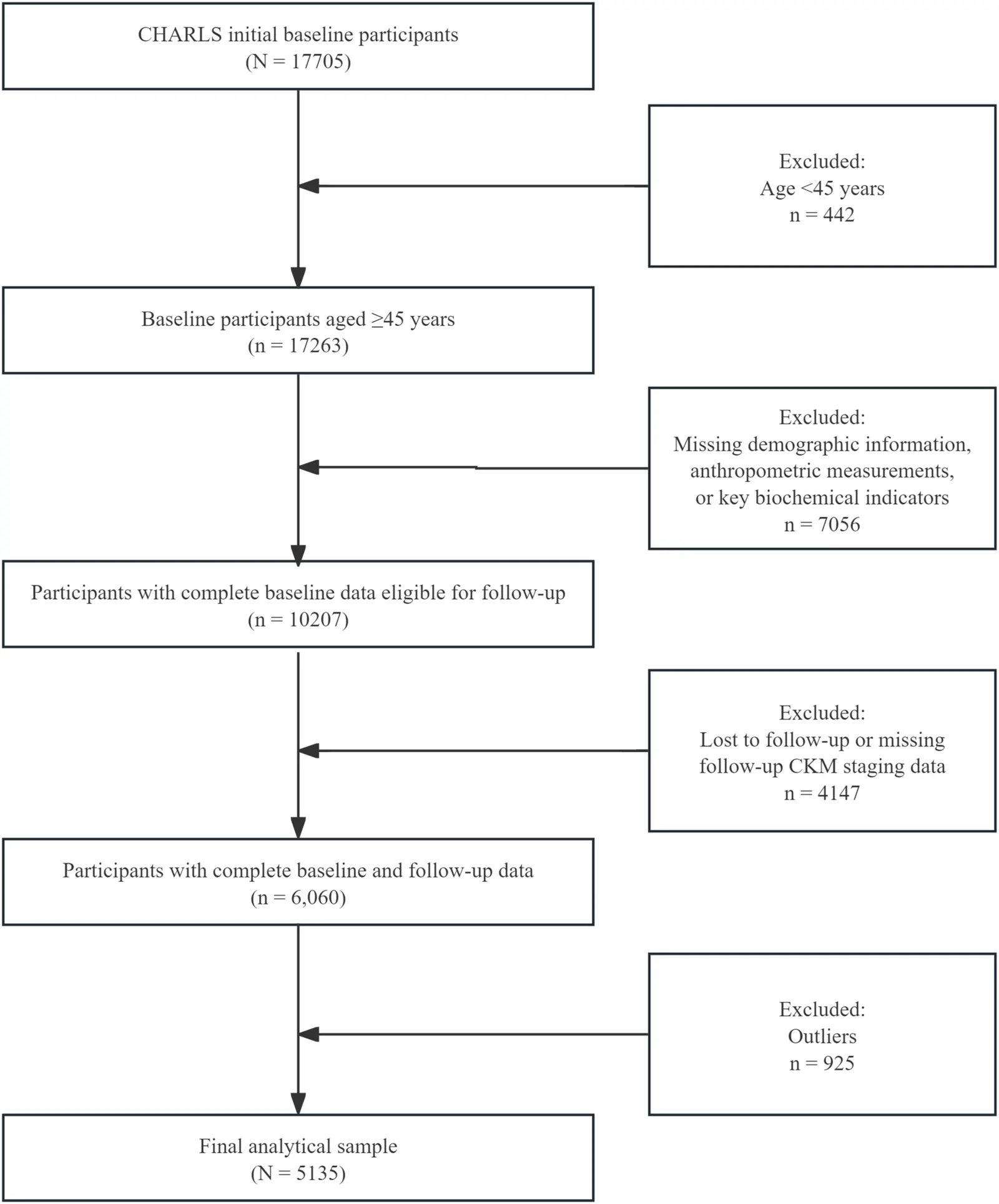
Flowchart of participant selection in the CHARLS study. CHARLS, China health and retirement longitudinal study; CKM, cardiovascular-kidney-metabolic syndrome.

### 2.2 Tyg index

The main exposure variable in this study was the TyG index. The TyG index is a simple indicator calculated from fasting triglycerides (TG) and fasting plasma glucose (FPG). It has been widely used to assess insulin resistance and metabolic abnormalities.

The TyG index was calculated as follows:

TyG=ln((TG×FPG)/2)

In the statistical analysis, the TyG index was included in the models both as a continuous variable and as a categorical variable. For the categorical analysis, participants were divided into T1, T2, and T3 groups according to the tertile distribution of the baseline TyG index, with the lowest tertile group (T1) used as the reference group.

### 2.3 Definition of CKM progression

CKM status at each survey wave was classified into stages 0−4 based on the AHA CKM staging framework and the variables available in CHARLS. Because not all components of the original framework were available, CKM stages were operationally defined using the available metabolic, kidney, and cardiovascular measures (1). Detailed operational criteria are provided in Supplementary Table S1. To improve the clinical interpretability of the model results and to focus on the transition of CKM from a relatively low-risk state to a high-risk state, and in accordance with guideline definitions, CKM stages 0−2 were combined and defined as the low-risk state in the main analysis, indicating that participants had not yet entered the stage of subclinical or clinical cardiovascular disease. CKM stages 3–4 were combined and defined as the high-risk state, indicating increased risk of subclinical cardiovascular disease or the presence of clinical cardiovascular disease. CKM progression was defined as transition from the low-risk state (CKM stages 0–2) to the high-risk state (CKM stages 3–4) during follow-up. CKM reversal was defined as transition from the high-risk state (CKM stages 3–4) to the low-risk state (CKM stages 0–2).

### 2.4 Covariates

Baseline covariates included age, sex, education, marriage, residence, smoking, alcohol, BMI, hypertension, hyperlipidemia, diabetes, hypertension medication, hyperlipidemia medication, and diabetes medication.

### 2.5 Statistical analysis

#### 2.5.1 Descriptive analysis

Continuous variables with a normal distribution were expressed as mean ± standard deviation (Mean ± SD), whereas continuous variables with a non-normal distribution were expressed as median and interquartile range (IQR). Categorical variables were presented as frequencies and percentages. This study used complete case analysis (9), excluding all observations with missing or abnormal values to ensure model consistency.

#### 2.5.2 Multistate markov model analysis

A continuous-time multistate Markov model was used to describe the dynamic transition process across different CKM stages and to estimate transition intensities, transition probabilities, and expected sojourn times between CKM states. The analysis was based on data from two CHARLS waves, namely 2011 (Wave 1) and 2015 (Wave 3). According to CKM staging results at baseline and follow-up waves, participants' health status at each observation time point was classified into five mutually exclusive states: CKM stage 0, CKM stage 1, CKM stage 2, CKM stage 3, and CKM stage 4. Each participant could occupy only one state at any given time point. Considering the clinical course of CKM, CKM stage 4 was defined as an absorbing state. Once participants entered CKM stage 4, they were no longer allowed to transition to any lower stage. For CKM stages 0–3, bidirectional transitions between different stages were allowed to reflect the dynamic process in which CKM progression and reversal may coexist. The corresponding five-state transition intensity matrix is shown below.

Let the stochastic process X(t) denote the state occupied by an individual at time t. If X(t) = r, the individual is in state r at time t. The instantaneous transition rate from state r to state s, namely the transition intensity, is defined as:

$$q_{rs}=\underset{\delta t\to 0}{\lim}\frac{P(X(t+\delta t)=s|X(t)=r)}{\delta t},\;r\neq s$$

All transition intensities constitute the transition intensity matrix Q = (qrs), which was specified as a 5 × 5 matrix in this study:

$$Q=\left(\begin{array}{ccccc} -(q_{01}+q_{02}+q_{03}+q_{04}) & q_{01} & q_{02} & q_{03} & q_{04}\\ q_{10} & -(q_{10}+q_{12}+q_{13}+q_{14}) & q_{12} & q_{13} & q_{14}\\ q_{20} & q_{21} & -(q_{20}+q_{21}+q_{23}+q_{24}) & q_{23} & q_{24}\\ q_{30} & q_{31} & q_{32} & -(q_{30}+q_{31}+q_{32}+q_{34}) & q_{34}\\ 0 & 0 & 0 & 0 & 0 \end{array}\right)$$

The model was based on the Markov assumption, indicating that the future CKM state depends only on the current state rather than the previous transition history. The model also assumed time-homogeneous transition intensities, indicating that the transition rates remained constant over the observation period and represented the average transition patterns during follow-up.

Based on the estimated transition intensity matrix, the state transition probability matrix at different follow-up times was calculated using the matrix exponential function P(t)=exp(Qt), thereby describing the dynamic transition patterns across CKM states. The expected sojourn time in each transient state was calculated as:

$$E(T_{s})=-\frac{1}{q_{ss}}$$

where q_ss_ denotes the diagonal element of the transition intensity matrix for state s. The diagonal element q_ss_ is equal to the negative sum of all transition intensities from state s to other states. The expected sojourn time therefore represents the mean duration that an individual is expected to remain in state s before transitioning to another state. Because CKM stage 4 was specified as an absorbing state, its expected sojourn time was not estimated.

#### 2.5.3 Simplified two-state markov model

To improve the clinical interpretability of the results, further assess the association between the TyG index and clinically meaningful CKM state transitions, reduce the number of model parameters, and improve the stability of model estimation, this study further constructed a simplified two-state Markov model. According to CKM staging, CKM stages 0–2 were combined and defined as the low-risk state, while CKM stages 3−4 were defined as the high-risk state. This model allowed bidirectional transitions between the two states. After adjustment for prespecified covariates, the associations of the TyG index with transition from the low-risk CKM state to the high-risk CKM state and from the high-risk CKM state to the low-risk CKM state were evaluated. Effect estimates were expressed as hazard ratios (HRs) and their 95% confidence intervals (CIs).

#### 2.5.4 Restricted cubic spline analysis

Restricted cubic spline analyses based on Cox proportional hazards models were performed to evaluate the dose-response associations of the baseline TyG index with CKM progression and reversal. Progression from the low-risk to the high-risk CKM state and reversal from the high-risk to the low-risk CKM state were defined as the respective outcomes. The models were adjusted for age, sex, education, marriage, residence, smoking, alcohol, BMI, hypertension, hyperlipidemia, diabetes, hypertension medication, hyperlipidemia medication, and diabetes medication.

#### 2.5.5 Sensitivity analyses

To assess the robustness of the findings, three sensitivity analyses were performed. First, the TyG index was included in the two-state Markov model as a continuous variable to evaluate the associations of each 1-unit increase and each 1-SD increase in TyG with CKM progression and reversal. Second, to examine whether the primary findings were influenced by participants with clinical CVD at baseline, the two-state Markov model was refitted after excluding participants with CKM stage 4 at baseline. The same definitions of the low-risk and high-risk CKM states and the same covariate adjustment strategy as those used in the primary analysis were applied. Third, a restricted five-state Markov model allowing transitions only between adjacent CKM stages was fitted to evaluate the robustness of the estimated CKM transition pattern to the specification of nonadjacent transitions. CKM stage 4 remained an absorbing state in the restricted model.

All statistical analyses were performed using R. The multistate Markov models were fitted using the msm package, and the Cox proportional hazards models and restricted cubic spline analyses were performed using the survival and rms packages. All tests were two-sided, and *P* < 0.05 was considered statistically significant.

## 3 Results

### 3.1 Baseline characteristics of the study population

A total of 5,135 participants were included in the study (Table 1). According to the tertile distribution of the baseline TyG index, participants were classified into the T1 group (low TyG, *n* = 1712), T2 group (moderate TyG, *n* = 1,712), and T3 group (high TyG, *n* = 1,711).

**Table 1:** Baseline characteristics of participants according to TyG tertiles.

Variable	Overall (*N* = 5,135)	T1 (*N* = 1,712)	T2 (*N* = 1,712)	T3 (*N* = 1,711)	*P*-value
Age, years	59.51 ± 8.94	59.48 ± 9.36	59.67 ± 8.80	59.39 ± 8.64	0.645
BMI, kg/m^2^	23.33 ± 3.40	22.26 ± 3.09	23.26 ± 3.32	24.47 ± 3.43	<0.001
Triglycerides, mg/dL	100.00 (72.57–138.95)	63.72 (54.87–73.46)	100.00 (89.39–110.62)	160.18 (138.06–195.59)	<0.001
Fasting glucose, mg/dL	100.98 (94.14–108.36)	96.66 (90.90–103.50)	100.98 (94.50–108.00)	104.94 (98.28–112.68)	<0.001
Height, m	1.58 ± 0.08	1.58 ± 0.08	1.58 ± 0.08	1.58 ± 0.08	0.122
Weight, kg	58.42 ± 10.28	55.93 ± 9.31	58.22 ± 10.30	61.11 ± 10.54	<0.001
Sex					
Female	2,728 (53.1%)	788 (46.0%)	896 (52.3%)	1,044 (61.0%)	<0.001
Male	2,407 (46.9%)	924 (54.0%)	816 (47.7%)	667 (39.0%)	
Marriage					
Married	5,082 (99.0%)	1,689 (98.7%)	1,699 (99.2%)	1,694 (99.0%)	0.235
Others	53 (1.0%)	23 (1.3%)	13 (0.8%)	17 (1.0%)	
Education					
Below primary school	2,430 (47.3%)	801 (46.8%)	812 (47.4%)	817 (47.7%)	0.613
Primary school	1,222 (23.8%)	407 (23.8%)	423 (24.7%)	392 (22.9%)	
Middle school	1,006 (19.6%)	349 (20.4%)	311 (18.2%)	346 (20.2%)	
High school or above	477 (9.3%)	155 (9.1%)	166 (9.7%)	156 (9.1%)	
Residence					
Rural	3,440 (67.0%)	1,231 (71.9%)	1,151 (67.2%)	1,058 (61.8%)	<0.001
Urban	1,695 (33.0%)	481 (28.1%)	561 (32.8%)	653 (38.2%)	
Smoking					
Never	3,105 (60.5%)	956 (55.8%)	1,027 (60.0%)	1,122 (65.6%)	<0.001
Current	1,597 (31.1%)	605 (35.3%)	548 (32.0%)	444 (25.9%)	
Former	433 (8.4%)	151 (8.8%)	137 (8.0%)	145 (8.5%)	
Alcohol					
No	3,426 (66.7%)	1,044 (61.0%)	1,156 (67.5%)	1,226 (71.7%)	<0.001
Yes	1,709 (33.3%)	668 (39.0%)	556 (32.5%)	485 (28.3%)	
Hypertension					
No	3,095 (60.3%)	1,180 (68.9%)	1,049 (61.3%)	866 (50.6%)	<0.001
Yes	2,040 (39.7%)	532 (31.1%)	663 (38.7%)	845 (49.4%)	
Diabetes					
No	4,747 (92.4%)	1,642 (95.9%)	1,592 (93.0%)	1,513 (88.4%)	<0.001
Yes	388 (7.6%)	70 (4.1%)	120 (7.0%)	198 (11.6%)	
Hyperlipidemia					
No	4,187 (81.5%)	1,595 (93.2%)	1,455 (85.0%)	1,137 (66.5%)	<0.001
Yes	948 (18.5%)	117 (6.8%)	257 (15.0%)	574 (33.5%)	
Hypertension medication					
No	4,960 (96.6%)	1,643 (96.0%)	1,655 (96.7%)	1,662 (97.1%)	0.166
Yes	175 (3.4%)	69 (4.0%)	57 (3.3%)	49 (2.9%)	
Hyperlipidemia medication					
No	4,989 (97.2%)	1,665 (97.3%)	1,666 (97.3%)	1,658 (96.9%)	0.737
Yes	146 (2.8%)	47 (2.7%)	46 (2.7%)	53 (3.1%)	
Diabetes medication					
No	4,783 (93.1%)	1,594 (93.1%)	1,594 (93.1%)	1,595 (93.2%)	0.989
Yes	352 (6.9%)	118 (6.9%)	118 (6.9%)	116 (6.8%)	
CKM stage					
Stage 0	359 (7.0%)	256 (15.0%)	98 (5.7%)	5 (0.3%)	<0.001
Stage 1	911 (17.7%)	470 (27.5%)	388 (22.7%)	53 (3.1%)	
Stage 2	1,662 (32.4%)	408 (23.8%)	500 (29.2%)	754 (44.1%)	
Stage 3	1,426 (27.8%)	381 (22.3%)	461 (26.9%)	584 (34.1%)	
Stage 4	777 (15.1%)	197 (11.5%)	265 (15.5%)	315 (18.4%)	

TyG, triglyceride-glucose index; BMI, body mass index; CKM, cardiovascular-kidney-metabolic syndrome.

The mean age of the study population was 59.51 ± 8.94 years, and 53.1% were women. Significant differences across TyG tertiles were observed in sex, residence, smoking, alcohol, BMI, weight, triglycerides, fasting glucose, CKM stage, and the prevalence of hypertension, diabetes, and hyperlipidemia (all *P* < 0.001). As TyG levels increased, BMI, weight, triglycerides, and fasting glucose progressively increased, along with the prevalence of hypertension, diabetes, and hyperlipidemia. The distribution of CKM stages also differed significantly across TyG tertiles. Participants in the T1 group had higher proportions of CKM stages 0–1, whereas those in the T3 group had higher proportions of CKM stages 2–4. In the T3 group, the proportions of CKM stages 2, 3, and 4 were 44.1%, 34.1%, and 18.4%, respectively. No significant differences were observed across TyG tertiles in age, marriage, education, height, hypertension medication, hyperlipidemia medication, or diabetes medication (all *P* > 0.05).

### 3.2 Dynamic transitions and transition intensities across CKM stages

At baseline, participants in CKM stages 0–4 accounted for 6.99%, 17.74%, 32.37%, 27.77%, and 15.13% of the study population, respectively (Supplementary Table S2). Bidirectional transitions among CKM stages 0–3 were observed between 2011 and 2015, whereas all participants with CKM stage 4 at baseline remained in stage 4 at follow-up. The observed proportions of participants remaining in their initial stages were 17.0%, 34.0%, 55.7%, and 71.8% for CKM stages 0, 1, 2, and 3, respectively. The observed proportion progressing to CKM stage 4 increased from 8.4% among participants initially in stage 0 to 13.9% among those initially in stage 3.

The estimated transition intensities are presented in Table 2. Among progression transitions, the highest intensity was observed from CKM stage 0 to stage 1 (0.382, 95% CI: 0.286–0.510), followed by the transition from stage 1 to stage 2 (0.220, 95% CI: 0.189–0.257). Among transitions to lower stages, the intensities from stage 1 to stage 0 and from stage 2 to stage 1 were 0.068 (95% CI: 0.046–0.101) and 0.061 (95% CI: 0.049–0.075), respectively, whereas transitions from stage 3 to lower stages were less frequent. The transition intensity to CKM stage 4 increased from 0.018 for participants in stages 0 and 1 to 0.032 for those in stage 2 and 0.038 for those in stage 3.

**Table 2:** Transition intensities among CKM stages.

Transition category	Transition							Transition intensity, year^−1^ (95% CI)
Progression								
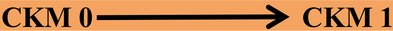								0.382 (0.286, 0.510)
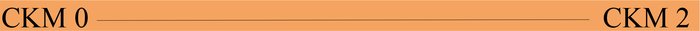								0.032 (0.004, 0.250)
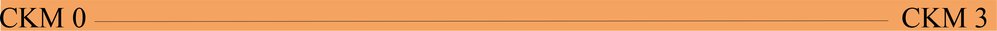								0.076 (0.047, 0.122)
	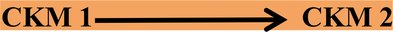							0.220 (0.189, 0.257)
	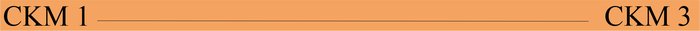							0.037 (0.023, 0.058)
			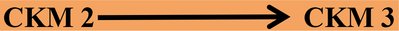					0.077 (0.068, 0.087)
Reversal								
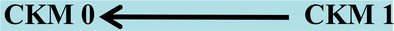								0.068 (0.046, 0.101)
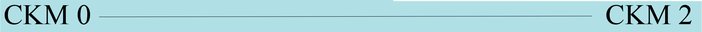								0.006 (0.002, 0.019)
	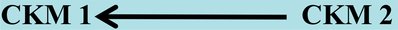							0.061 (0.049, 0.075)
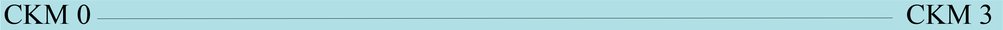								0.001 (0.000, 0.012)
	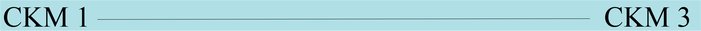							0.012 (0.007, 0.019)
			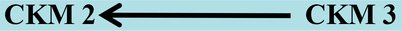					0.039 (0.032, 0.047)
Outcome								
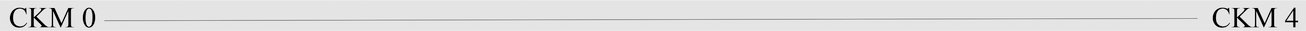								0.018 (0.006, 0.052)
	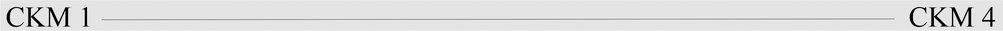							0.018 (0.010, 0.030)
			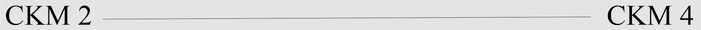					0.032 (0.027, 0.039)
					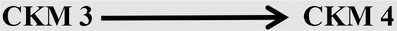			0.038 (0.033, 0.045)

CKM, cardiovascular-kidney-metabolic syndrome; CI, confidence interval.

### 3.3 Transition probabilities across CKM stages

The model-predicted transition probabilities across CKM stages are presented in Figure 2 and Supplementary Figure S1. As follow-up time increased, the probability of remaining in the initial CKM stage gradually decreased. Among participants initially in CKM stages 0, 1, 2, and 3, the probabilities of remaining in the same stage decreased from 61.0%, 72.4%, 84.5%, and 91.6% at 1 year to 17.0%, 34.0%, 55.7%, and 71.8% at 4 years, respectively. Meanwhile, the probabilities of transitioning to higher CKM stages generally increased over time. The model-predicted probabilities of being in CKM stage 4 at 4 years were 8.3%, 8.8%, 12.0%, and 13.9% among participants initially in CKM stages 0, 1, 2, and 3, respectively, compared with approximately 1.9%–3.7% at 1 year.

**Figure 2:**
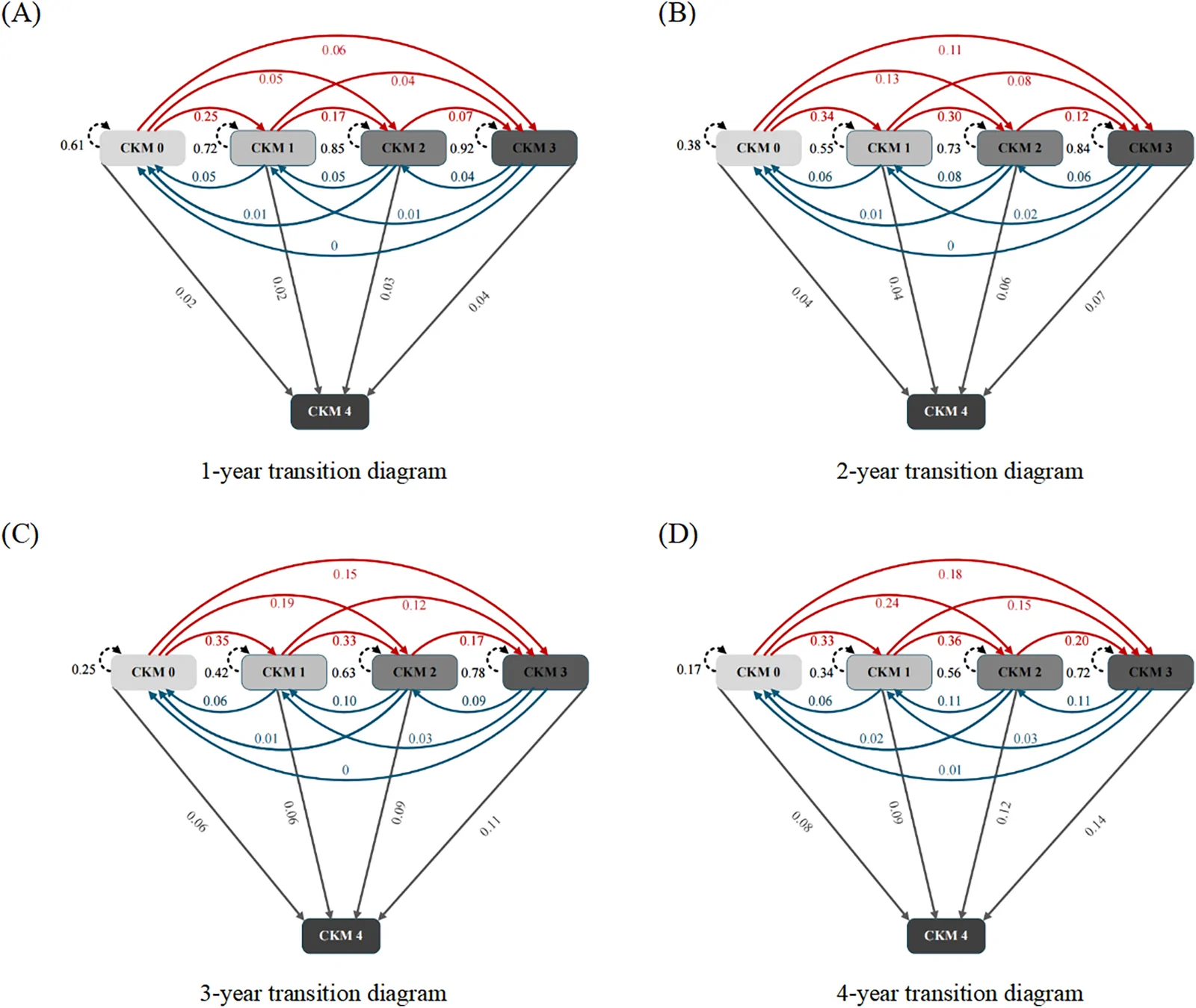
Predicted state transition probabilities among CKM stages. **(A)** Predicted state transition probabilities among CKM stages at 1 year. **(B)** Predicted state transition probabilities among CKM stages at 2 years. **(C)** Predicted state transition probabilities among CKM stages at 3 years. **(D)** Predicted state transition probabilities among CKM stages at 4 years. Arrows indicate possible transition directions between CKM stages, and numbers represent the corresponding predicted transition probabilities derived from the continuous-time Markov model. Red arrows indicate transitions toward higher CKM stages, blue arrows indicate transitions toward lower CKM stages, and gray arrows indicate transitions to CKM stage 4, which was treated as an absorbing state. CKM, cardiovascular-kidney-metabolic syndrome.

The expected sojourn times in the transient CKM stages are presented in Supplementary Table S3. The expected sojourn times in CKM stages 0, 1, 2, and 3 were 1.97, 2.92, 5.72, and 10.64 years, respectively, indicating progressively longer expected durations with advancing CKM stage.

### 3.4 Association between TyG and CKM state transitions based on the two-state (2 × 2) markov model

#### 3.4.1 CKM state transitions across TyG groups

In the two-state analysis, 2,932 participants were in the low-risk state (CKM stages 0–2) at baseline. Among them, 2,089 (71.2%) remained in the low-risk state, whereas 843 (28.8%) progressed to the high-risk state during follow-up. Among the 2,203 participants in the high-risk state (CKM stages 3–4) at baseline, 204 (9.3%) reversed to the low-risk state, whereas 1,999 (90.7%) remained in the high-risk state (Supplementary Table S4).

The observed transition proportions across TyG groups are presented in Supplementary Figure S2. The proportions progressing from the low-risk state to the high-risk state were 26.6%, 29.3%, and 31.0% in the T1, T2, and T3 groups, respectively. The corresponding proportions reversing from the high-risk state to the low-risk state were 9.2%, 7.9%, and 10.5%, respectively.

#### 3.4.2 CKM progression

The adjusted associations of TyG tertiles with CKM progression and reversal are presented in the forest plot in Figure 3. After adjustment for prespecified covariates, the association between the T2 group and progression from the low-risk state to the high-risk state was not statistically significant compared with the T1 group (HR = 1.15, 95% CI: 0.96–1.37, *P* = 0.131). In contrast, participants in the T3 group had a significantly higher risk of CKM progression (HR = 1.35, 95% CI: 1.11–1.65, *P* = 0.002). RCS analysis showed a significant overall association between the TyG index and CKM progression (*P* for overall = 0.005), with no evidence of a nonlinear relationship (*P* for nonlinearity = 0.500) (Supplementary Figure S3).

**Figure 3:**
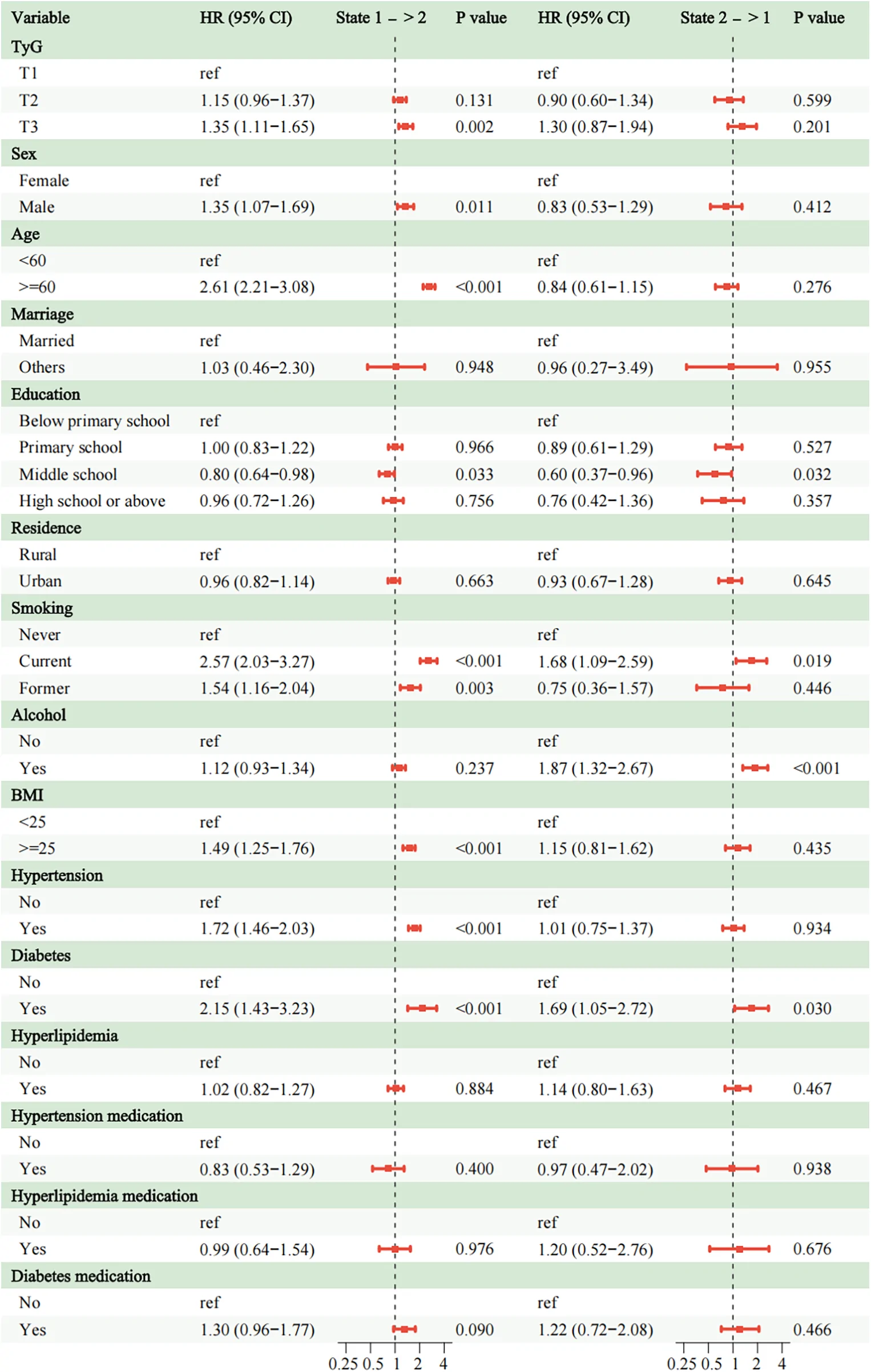
Association of TyG with bidirectional transitions between low-risk and high-risk CKM states: a forest plot based on a two-state Markov model. State 1 → 2 represents transition from the low-risk state to the high-risk state, whereas State 2 → 1 represents reversal from the high-risk state to the low-risk state. Red dots indicate hazard ratios (HRs), and horizontal lines indicate 95% confidence intervals (CIs). The model was adjusted for age, sex, marital status, education level, residence, smoking status, alcohol consumption, BMI, hypertension, diabetes, hyperlipidemia, and hypertension medication, hyperlipidemia medication, and diabetes medication. TyG, triglyceride-glucose index; CKM, cardiovascular-kidney-metabolic syndrome; State 1, low-risk state defined as CKM stages 0–2; State 2, high-risk state defined as CKM stages 3–4; HR, hazard ratio; CI, confidence interval; BMI, body mass index.

#### 3.4.3 CKM reversal

The forest plot showed no significant association between TyG levels and reversal from the high-risk state to the low-risk state. Compared with the T1 group, the HR was 0.90 (95% CI: 0.60–1.34, *P* = 0.599) for the T2 group and 1.30 (95% CI: 0.87–1.94, *P* = 0.201) for the T3 group (Figure 3). RCS analysis also showed no significant overall association between the TyG index and CKM reversal (*P* for overall = 0.266), with no evidence of a nonlinear relationship (*P* for nonlinearity = 0.220) (Supplementary Figure S4).

#### 3.4.4 Sojourn time in low-risk and high-risk CKM states across TyG groups

The model-estimated expected sojourn times are presented in Supplementary Table S5. The expected sojourn times in the low-risk state were 12.13, 10.90, and 9.98 years for the T1, T2, and T3 groups, respectively, showing a progressive decrease with increasing TyG levels. The corresponding expected sojourn times in the high-risk state were 35.24, 40.74, and 29.61 years, respectively, without a consistent monotonic pattern across TyG groups.

### 3.5 Sensitivity analyses

Sensitivity analyses generally supported the primary findings. When TyG was modeled as a continuous variable, each 1-unit and 1-SD increase was associated with a higher risk of CKM progression, with HRs of 1.44 (95% CI: 1.21–1.72) and 1.19 (95% CI: 1.09–1.29), respectively, whereas no significant associations were observed with CKM reversal (Supplementary Table S6). After excluding participants with CKM stage 4 at baseline, the association between the highest TyG tertile and CKM progression remained significant (HR = 1.36, 95% CI: 1.06–1.73), while the associations with reversal remained nonsignificant (Supplementary Table S7). The restricted adjacent-transition model showed a broadly similar pattern of bidirectional transitions and gradual progression across CKM stages (Supplementary Tables S8A and S8B).

## 4 Discussion

This study was based on the national longitudinal CHARLS cohort and used a continuous-time multistate Markov model to characterize the dynamic transitions across different stages of CKM syndrome. A two-state Markov model was further applied to examine the associations of the TyG index with transitions between low- and high-risk CKM states. The results showed that CKM stages exhibited clear dynamic evolution, with more active transitions at earlier stages, longer sojourn times at higher stages, and an overall tendency to accumulate in CKM stage 4. In the two-state model, compared with participants in the lowest TyG tertile, those in the second tertile did not have a significantly increased risk of CKM progression, whereas those in the highest tertile had a 35% higher risk of transitioning from the low-risk to the high-risk state. Restricted cubic spline analysis further demonstrated a significant linear positive association between the TyG index and the risk of CKM progression. In contrast, no significant association was observed between the TyG index and transitions from the high-risk to the low-risk CKM state.

The associations of the TyG index with CKM transitions remained broadly consistent when TyG was analyzed as a continuous variable and when participants with CKM stage 4 at baseline were excluded. Separately, the restricted adjacent-transition model supported the overall pattern of gradual progression across CKM stages. The estimated sojourn times across TyG groups also showed that participants with higher TyG levels spent progressively less time in the low-risk CKM state. These findings suggest that a higher TyG level may be associated with an increased likelihood of CKM progression from the low-risk to the high-risk state and may provide complementary information for identifying individuals at elevated risk of CKM progression.

CKM was recently proposed by the AHA to emphasize the complex pathological interrelationships among metabolic abnormalities, chronic kidney damage, and CVD (1, 10). The AHA conceptualizes CKM as a continuum extending from the absence of risk factors through adipose tissue dysfunction and metabolic risk factors to subclinical and clinical CVD, rather than as a single disease state. Conventional single-endpoint Cox models are often unable to fully capture the intermediate stages experienced by individuals before the onset of CVD. Accordingly, this study applied a multistate Markov model incorporating CKM stages 0–4 into a unified longitudinal analytical framework, allowing the simultaneous estimation of instantaneous transition intensities, expected sojourn times, and long-term transition probabilities across CKM stages, thereby more closely reflecting the natural course of CKM. Previous studies have demonstrated that multistate models are particularly suitable for chronic diseases characterized by stagewise progression, intermittently observed states, and the possibility of bidirectional transitions (11–13).

We observed bidirectional transitions among CKM stages 0–3, indicating that CKM does not invariably progress in a single direction but may remain stable, progress, or transition to a lower stage during follow-up. The transition intensities from CKM stage 0 to stage 1 and from stage 1 to stage 2 were relatively high, suggesting that the early stages of CKM are highly dynamic. Early-stage CKM primarily involves adipose tissue dysfunction, insulin resistance, and the gradual accumulation of metabolic risk factors, including abnormalities in glucose, lipid, and blood pressure levels (1, 14). At these stages, metabolic status may be more readily influenced by body weight, lifestyle, disease control, and health management, resulting in more frequent changes in CKM status. In contrast, as CKM stage increased, the expected sojourn time in the corresponding state became progressively longer, whereas the transition intensities toward lower stages generally decreased. Persistent disturbances in glucose and lipid metabolism, chronic low-grade inflammation, endothelial dysfunction, kidney damage, and myocardial structural alterations may collectively contribute to the maintenance and further progression of advanced CKM states (3, 15–17). These findings are consistent with the conceptualization of CKM as a continuous disease process, in which the long-term accumulation of metabolic risk factors and organ damage may gradually move individuals toward higher levels of kidney and cardiovascular risk (1, 10). Furthermore, the restricted five-state model allowing only adjacent transitions showed a similar overall pattern of gradual progression across CKM stages, supporting the transition pattern observed in the primary analysis.

Among the factors associated with CKM progression, the TyG index has received increasing attention in recent years as a simple surrogate marker of insulin resistance in the risk assessment of metabolic and cardiovascular diseases. The TyG index is calculated from fasting triglyceride and fasting glucose levels (4) and can simultaneously reflect abnormalities in glucose and lipid metabolism. Its advantages include convenient measurement, low cost, and suitability for large-scale population screening. Previous studies have shown that the TyG index is closely associated with the development of metabolic syndrome (5), type 2 diabetes (6), and atherosclerotic CVD (7). In addition, prospective studies and systematic reviews have demonstrated associations between elevated TyG levels and coronary heart disease, cardiovascular events, and adverse cardiovascular outcomes (18, 19). Collectively, these findings suggest that the TyG index is not merely a metabolic marker but may also reflect the continuum of pathological changes from metabolic abnormalities to cardiovascular damage.

Previous studies have largely focused on diabetes, coronary heart disease, stroke, or cardiovascular events as single outcomes, with limited evaluation of the relationship between the TyG index and changes across different disease states within the CKM continuum. A recent study also based on CHARLS found that higher TyG levels were associated with an increased risk of CKM stage progression (20). The present study further showed that higher TyG levels were primarily associated with progression from the low-risk to the high-risk CKM state. Compared with previous studies, this study used a multistate Markov model to characterize the dynamic transitions across CKM stages 0–4 and separately evaluated the associations of the TyG index with CKM progression and reversal.

The association between the TyG index and CKM progression may be largely attributable to insulin resistance and the accompanying constellation of metabolic abnormalities. Insulin resistance is considered a key pathophysiological link connecting obesity, disturbances in glucose and lipid metabolism, CKD, and CVD (3). In the presence of insulin resistance, hyperinsulinemia, lipid metabolic dysfunction, chronic low-grade inflammation, oxidative stress, and endothelial dysfunction may occur concurrently (21, 22), thereby contributing to atherosclerosis, glomerular hyperfiltration, tubular injury, and myocardial remodeling (23–25).

Because the TyG index integrates information on both fasting glucose and triglycerides, it may provide a more comprehensive reflection of glucose and lipid metabolic burden than either measure alone. Among individuals in CKM stages 0–2, a higher TyG level may indicate more pronounced insulin resistance, hyperglycemia, hypertriglyceridemia, and lipotoxicity, thereby increasing the likelihood of progression to a higher CKM stage. Prospective cohort studies have shown that higher baseline TyG levels, longitudinal increases in TyG, and greater TyG variability are associated with an increased risk of cardiovascular events after adjustment for conventional cardiovascular risk factors (26–29). Moreover, the expected sojourn time in the low-risk CKM state decreased progressively with increasing TyG levels, consistent with the observed increase in CKM progression risk. In contrast, the expected sojourn time in the high-risk CKM state did not show a similar pattern across TyG tertiles, with the shortest estimate observed in the T3 group. Because expected sojourn time is primarily determined by the transition intensity out of the current state (12), the duration of the high-risk state reflects subsequent transitions after entering that state rather than progression from the low-risk to the high-risk state itself. In this study, the observed proportion of reversal from the high-risk to the low-risk state was numerically higher in the T3 group, which was directionally consistent with its shorter expected sojourn time in the high-risk state. However, the adjusted association between TyG and CKM reversal was not statistically significant, suggesting that differences in the duration of the high-risk state may also be influenced by underlying disease burden, treatment changes, and other clinical factors.

In contrast to CKM progression, no significant association was observed between the TyG index and reversal from the high-risk to the low-risk CKM state. Similar findings were obtained when the TyG index was modeled as a continuous variable and after participants with CKM stage 4 at baseline were excluded, suggesting that the null association was not entirely driven by participants with established clinical CVD at baseline. This finding may indicate that the TyG index, which primarily reflects insulin resistance and glucose-lipid metabolic burden, is more closely related to CKM progression than to reversal. Reversal from the high-risk to the low-risk state may depend on a broader range of clinical factors that are not fully captured by the TyG index alone.

The differential associations of the TyG index with CKM progression and reversal suggest that the determinants of CKM deterioration and improvement may not be identical. Because the TyG index primarily reflects insulin resistance and glucose-lipid metabolic burden, its association may be more pronounced for progression from the low-risk to the high-risk state, whereas changes at more advanced CKM stages may be influenced to a greater extent by pre-existing disease burden and comprehensive clinical management.

These findings also have potential clinical and public health implications. The TyG index is derived from routinely measured fasting glucose and triglyceride levels, is simple to calculate, and is relatively inexpensive, making it readily applicable in primary care and large-scale population health management. Among individuals in CKM stages 0–2, higher TyG levels may indicate an increased risk of progression to CKM stages 3–4 and may help identify those who require further comprehensive assessment of cardiovascular, kidney, and metabolic risk. The TyG index may complement existing CKM risk assessment measures but should not replace conventional clinical information, including blood pressure, glucose, lipid levels, kidney function, and a history of CVD. Middle-aged and older adults with elevated TyG levels should therefore be evaluated comprehensively by considering body weight, blood pressure, glucose, lipid levels, kidney function, and pre-existing diseases. Because this was an observational analysis, the identified associations between the TyG index and CKM state transitions do not establish that lowering the TyG index itself would directly prevent CKM progression. Accordingly, the primary clinical value of these findings lies in risk identification and stratification rather than in defining specific treatment thresholds or intervention strategies. For individuals already at more advanced CKM stages, comprehensive management should continue to account for disease severity, established organ damage, and current clinical treatment.

This study has several strengths. First, CKM stages 0–4 were incorporated into a unified continuous-time multistate Markov model, which captured not only the maintenance of the current state and progression to higher stages but also transitions to lower stages, thereby providing a more comprehensive representation of the dynamic evolution of CKM than conventional single-endpoint analyses. Second, a two-state Markov model was further constructed by defining CKM stages 0–2 as the low-risk state and stages 3–4 as the high-risk state, allowing the association of the TyG index with clinically meaningful bidirectional transitions to be evaluated. In addition, the robustness of the associations between the TyG index and CKM transitions was examined by modeling TyG as a continuous variable and by excluding participants with CKM stage 4 at baseline, whereas the restricted adjacent-transition model assessed the stability of the overall CKM transition pattern under a different model structure.

Several limitations should also be acknowledged. Because this study was based on observational cohort data, residual confounding cannot be completely excluded despite adjustment for a range of demographic, lifestyle, and clinical factors. Unmeasured or incompletely measured factors, including dietary patterns, access to healthcare, long-term disease management, and the severity and duration of kidney and cardiovascular damage, may have influenced both TyG levels and changes in CKM status. In addition, the analysis adjusted only for hypertension medication, hyperlipidemia medication, and diabetes medication at baseline and did not account for changes in treatment during follow-up. Initiation, discontinuation, or modification of these medications may affect glucose and lipid levels, blood pressure, and TyG levels and may also influence CKM state transitions. Therefore, reliance on baseline medication information alone may not fully reflect treatment status throughout follow-up and may have introduced residual confounding. In addition, the analysis primarily relied on baseline TyG levels and did not account for longitudinal changes in TyG during follow-up. Because TyG may be influenced by lifestyle, disease progression, and pharmacological treatment, a single baseline measurement may not fully reflect the long-term metabolic burden over the entire follow-up period. Among participants whose TyG levels changed substantially during follow-up, reliance on the baseline value alone may also have affected the estimated association between TyG and CKM state transitions. Incorporating repeated TyG measurements may yield effect estimates and association patterns that differ from those based on a single baseline measurement. Future studies incorporating repeated TyG assessments may help further validate the present findings and provide a more comprehensive evaluation of the relationship between long-term changes in TyG and CKM state transitions. Third, CKM status was assessed only at the 2011 and 2015 waves, and the exact timing of state transitions could therefore not be directly determined. Short-term changes and intermediate states occurring between the two assessments may also have been missed. For nonadjacent stage changes observed between the two time points, the available data could not distinguish whether these represented direct transitions or gradual changes through one or more unobserved intermediate stages. Under this panel observation structure, the transition intensities and expected sojourn times estimated by the continuous-time Markov model were not based on directly observed transition times or state durations, but were inferred from changes in CKM status between the two assessments under the Markov and time-homogeneity assumptions. The relatively long interval between assessments may therefore have reduced the temporal precision with which CKM transition processes and state durations were characterized. Fourth, CKM stages were operationally defined using the metabolic, kidney, and cardiovascular measures available in CHARLS. The lack of urinary albumin-to-creatinine ratio and detailed measures of subclinical CVD prevented full implementation of the original AHA CKM staging framework and may have resulted in some degree of stage misclassification. Such potential misclassification may have affected the accuracy of CKM stage assignment and, consequently, the estimated transition patterns across CKM stages. In addition, because the present study used an operational CKM staging approach based on variables available in CHARLS, caution is warranted when generalizing these findings to populations in which the complete AHA CKM staging framework can be implemented. This study was conducted among Chinese adults aged 45 years and older, whose demographic characteristics, CKM burden, and patterns of clinical management may differ from those of other populations. Therefore, the association between TyG and CKM state transitions, as well as the magnitude of the observed associations, requires further validation in younger populations, other racial or ethnic groups, and healthcare systems with different CKM management practices. Fifth, the continuous-time Markov model relies on the Markov and time-homogeneity assumptions, whereby future states depend only on the current state and transition intensities are assumed to remain constant over the observation period. In practice, CKM evolution may also be influenced by prior disease history, repeated state changes, time-varying risk factors, and treatment, and the model may therefore simplify the underlying disease process. Finally, the limited number of events for some specific CKM transition pathways limited the ability to obtain stable estimates of the associations between TyG and individual transitions in the five-state model. The two-state model was therefore used to evaluate the overall associations of the TyG index with CKM progression and reversal. In addition, reversal from the high-risk to the low-risk CKM state was relatively infrequent, and the corresponding effect estimates had wide confidence intervals, indicating limited precision in the reversal analysis. Therefore, although no statistically significant association was observed between TyG and CKM reversal, a potential association cannot be completely excluded. Future longitudinal studies with larger sample sizes, more reversal events, more frequent follow-up, repeated TyG measurements, urinary albumin, repeated assessments of kidney and cardiovascular damage, and detailed treatment-related information are needed to evaluate specific transition pathways across CKM stages more precisely and further clarify the relationship between TyG and CKM reversal.

## Conclusion

In conclusion, CKM exhibited a clear dynamic evolutionary pattern, with more active transitions at earlier stages and longer expected sojourn times at more advanced stages. Higher TyG levels were associated with progression from the low-risk to the high-risk CKM state, whereas no significant association was observed with CKM reversal. The TyG index may provide simple complementary information for identifying individuals at increased risk of CKM progression, although its clinical application should be integrated with comprehensive assessment of metabolic, kidney, and cardiovascular factors.

## Data Availability

Publicly available datasets were analyzed in this study. The CHARLS datasets are available on the CHARLS website: http://charls.pku.edu.cn/en.
