# Supplementary material for: Association of the triglyceride-glucose index with dynamic bidirectional transitions of cardiovascular-kidney-metabolic syndrome: a multistate Markov model study based on CHARLS
Source: Front Med (Lausanne). 2026 Sep 28;13:1912039. doi: 10.3389/fmed.2026.1912039 (PMC13654756; doi:10.3389/fmed.2026.1912039)
Supplement: Supplementary file 1 [file Datasheet1.docx]

**Supplementary Materials**

| **Item** | **Title** | **Page** |
| --- | --- | --- |
| Table S1 | Definitions of CKM stages in CHARLS | 1 |
| Table S2 | Distribution of CKM stage transitions and cumulative progression to CKM stage 4 from 2011 to 2015 | 2 |
| Table S3 | Expected sojourn times in each transient CKM state, years | 3 |
| Table S4 | Transition distribution between low-risk and high-risk CKM states in the two-state Markov model | 3 |
| Table S5 | Expected sojourn times in low-risk and high-risk CKM states according to TyG tertiles, years | 3 |
| Table S6 | Sensitivity analysis of the association between continuous TyG index and bidirectional CKM transitions | 3 |
| Table S7 | Sensitivity analysis of the associations between TyG tertiles and bidirectional CKM transitions after excluding participants with CKM stage 4 at baseline | 3 |
| Table S8A | Transition intensity matrix from the restricted adjacent-transition five-state Markov model | 4 |
| Table S8B | Four-year predicted transition probabilities from the restricted five-state Markov model | 4 |
| Figure S1 | Predicted state transition probability heatmaps across CKM stages at 1, 2, 3, and 4 years | 5 |
| Figure S2 | Four-year observed transition proportions between low-risk and high-risk CKM states according to TyG tertiles | 6 |
| Figure S3 | Dose–response relationship between the TyG index and the risk of CKM progression from the low-risk to the high-risk state | 7 |
| Figure S4 | Dose–response relationship between the TyG index and the risk of CKM reversal from the high-risk to the low-risk state | 8 |

**Table S1.** Definitions of CKM Stages in the CHARLS

| **CKM Stage** | **Inclusion Requirement** | **Diagnostic Criteria** |
| --- | --- | --- |
| **Stage 0** | All criteria are met | BMI <23 kg/m²; waist circumference <80 cm in women or <90 cm in men; fasting blood glucose <100 mg/dL and HbA1c <5.7%, without self-reported diabetes or use of insulin or oral antidiabetic medication; SBP <130 mmHg and DBP <80 mmHg, without self-reported hypertension or use of antihypertensive medication; HDL-C ≥50 mg/dL in women or ≥40 mg/dL in men; triglycerides <150 mg/dL; eGFR ≥60 mL/min/1.73 m² without self-reported CKD; and no clinical CVD |
| **Stage 1** | Any of the following criteria was met, without meeting the criteria for stages 2–4 | Overweight or obesity: BMI ≥23 kg/m²; abdominal obesity: waist circumference ≥80 cm in women or ≥90 cm in men; or prediabetes: fasting blood glucose 100–124 mg/dL or HbA1c 5.7%–6.4%, without self-reported diabetes or use of insulin or oral antidiabetic medication |
| **Stage 2** | Any of the five criteria is met | Hypertriglyceridemia: triglycerides ≥135 mg/dL; hypertension: SBP ≥130 mmHg, DBP ≥80 mmHg, self-reported hypertension, or use of antihypertensive medication; diabetes: fasting blood glucose ≥125 mg/dL, HbA1c ≥6.5%, self-reported diabetes, or use of insulin or oral antidiabetic medication; metabolic syndrome; or CKD: eGFR 30–59 mL/min/1.73 m² or self-reported CKD |
| **Stage 3** | Any of the two criteria is met | Severe kidney dysfunction: eGFR <30 mL/min/1.73 m²; or high predicted cardiovascular risk: Framingham-predicted 10-year CVD risk ≥20% |
| **Stage 4** | The criterion is met | Self-reported physician-diagnosed heart disease, including angina, myocardial infarction, congestive heart failure, or other heart problems; or self-reported physician-diagnosed stroke |

CKM stages were assigned hierarchically according to the highest stage for which an individual met the criteria. The definitions were operationalized according to the metabolic, kidney, and cardiovascular variables available in CHARLS. Because urinary albumin-to-creatinine ratio and detailed measures of subclinical CVD were unavailable, the complete KDIGO risk classification combining eGFR and albuminuria and the original AHA CKM staging criteria could not be fully reproduced. Metabolic syndrome was defined as the presence of at least three of the following five components: (1) waist circumference ≥80 cm in women or ≥90 cm in men; (2) HDL-C <50 mg/dL in women or <40 mg/dL in men; (3) triglycerides ≥150 mg/dL; (4) SBP ≥130 mmHg, DBP ≥80 mmHg, or use of antihypertensive medication; and (5) fasting blood glucose ≥100 mg/dL. CKM, cardiovascular-kidney-metabolic; CHARLS, China Health and Retirement Longitudinal Study; BMI, body mass index; SBP, systolic blood pressure; DBP, diastolic blood pressure; HDL-C, high-density lipoprotein cholesterol; HbA1c, glycated hemoglobin; CKD, chronic kidney disease; eGFR, estimated glomerular filtration rate; CVD, cardiovascular disease; KDIGO, Kidney Disease: Improving Global Outcomes.

**Table S2.** Distribution of CKM stage transitions and cumulative progression to CKM stage 4 from 2011 to 2015

| **Baseline CKM stage** | **N** | **→ CKM 0** | **→ CKM 1** | **→ CKM 2** | **→ CKM 3** | **→ CKM 4** |
| --- | --- | --- | --- | --- | --- | --- |
| **CKM 0** | 359(6.99%) | 61 (17.00%) | 118 (32.90%) | 86 (24.00%) | 64 (17.80%) | 30 (8.40%) |
| **CKM 1** | 911(17.74%) | 55 (6.00%) | 310 (34.00%) | 330 (36.20%) | 136 (14.90%) | 80 (8.80%) |
| **CKM 2** | 1662(32.37%) | 28 (1.70%) | 175 (10.50%) | 926 (55.70%) | 334 (20.10%) | 199 (12.00%) |
| **CKM 3** | 1426(27.77%) | 6 (0.40%) | 47 (3.30%) | 151 (10.60%) | 1024 (71.80%) | 198 (13.90%) |
| **CKM 4** | 777(15.13%) | 0 (0.00%) | 0 (0.00%) | 0 (0.00%) | 0 (0.00%) | 777 (100.00%) |

CKM, cardiovascular-kidney-metabolic syndrome.

**Table S3.** Expected sojourn time of each transient CKM state (year)

| **State** | **estimates** |
| --- | --- |
| CKM 0 | 1.97 |
| CKM 1 | 2.92 |
| CKM 2 | 5.72 |
| CKM 3 | 10.64 |

CKM, cardiovascular-kidney-metabolic syndrome.

**Table S4.** Transition distribution between low-risk and high-risk CKM states in the two-state Markov model

| **Initial-State** | **Final-State** | **N** | **Percentage** |
| --- | --- | --- | --- |
| CKM 0-2 | CKM 0-2 (Stable) | 2089 | 71.2% |
| CKM 0-2 | CKM 3-4 (Progression) | 843 | 28.8% |
| CKM 3-4 | CKM 0-2 (Reversal) | 204 | 9.3% |
| CKM 3-4 | CKM 3-4 (Stable) | 1999 | 90.7% |

CKM, cardiovascular-kidney-metabolic syndrome.

**Table S5.** Expected sojourn time in low-risk and high-risk CKM states according to TyG tertiles (year)

| **State** | **T1** | **T2** | **T3** |
| --- | --- | --- | --- |
| CKM 0-2 | 12.13 | 10.90 | 9.98 |
| CKM 3-4 | 35.24 | 40.74 | 29.61 |

TyG, triglyceride-glucose index; CKM, cardiovascular-kidney-metabolic syndrome.

**Table S6.** Sensitivity analysis of the association between continuous TyG index and bidirectional CKM transitions

| **Analysis** | **Direction** | **HR (95% CI)** | ***P*-Value** |
| --- | --- | --- | --- |
| TyG as continuous (per 1 unit) | Progression (1->2) | 1.44 (1.21-1.72) | <0.001 |
| TyG as continuous (per 1 unit) | Reversal (2->1) | 1.41 (0.98-2.02) | 0.062 |
| TyG as continuous (per 1 SD) | Progression (1->2) | 1.19 (1.09-1.29) | <0.001 |
| TyG as continuous (per 1 SD) | Reversal (2->1) | 1.18 (0.99-1.40) | 0.062 |

TyG, triglyceride-glucose index; CKM, cardiovascular-kidney-metabolic syndrome;SD, standard deviation.

**Table S7.** Sensitivity analysis of the associations between TyG tertiles and bidirectional CKM transitions after excluding participants with CKM stage 4 at baseline

| **TyG tertile** | **Progression HR (95% CI)** | **P-value** | **Reversal HR (95% CI)** | **P-value** |
| --- | --- | --- | --- | --- |
| T1 | Reference |  | Reference |  |
| T2 | 1.10 (0.89–1.36) | 0.396 | 0.81 (0.52–1.27) | 0.361 |
| T3 | 1.36 (1.06–1.73) | 0.015 | 1.10 (0.69–1.74) | 0.696 |

TyG, triglyceride-glucose index; HR, hazard ratio.

**Table S8A**. Transition intensity matrix from the restricted adjacent-transition five-state Markov model

| **From** | **CKM 0** | **CKM 1** | **CKM 2** | **CKM 3** | **CKM 4** |
| --- | --- | --- | --- | --- | --- |
| CKM 0 | -0.686 | 0.686 | 0 | 0 | 0 |
| CKM 1 | 0.127 | -0.498 | 0.372 | 0 | 0 |
| CKM 2 | 0 | 0.104 | -0.263 | 0.159 | 0 |
| CKM 3 | 0 | 0 | 0.062 | -0.144 | 0.082 |
| CKM 4 | 0 | 0 | 0 | 0 | 0 |

CKM, cardiovascular-kidney-metabolic syndrome.

**Table S8B**. Four-year predicted transition probabilities from the restricted five-state Markov model

| **Initial CKM state** | **CKM 0** | **CKM 1** | **CKM 2** | **CKM 3** | **CKM 4** |
| --- | --- | --- | --- | --- | --- |
| CKM 0 | 0.135 | 0.377 | 0.373 | 0.104 | 0.011 |
| CKM 1 | 0.069 | 0.295 | 0.444 | 0.169 | 0.024 |
| CKM 2 | 0.019 | 0.124 | 0.477 | 0.316 | 0.065 |
| CKM 3 | 0.002 | 0.018 | 0.123 | 0.603 | 0.253 |
| CKM 4 | 0 | 0 | 0 | 0 | 1 |

CKM, cardiovascular-kidney-metabolic syndrome.

**Figure S1. Predicted state transition probability heatmaps across CKM stages over 1-, 2-, 3-, and 4-year time horizons.** (A) Predicted state transition probability heatmap at 1 year. (B) Predicted state transition probability heatmap at 2 years. (C) Predicted state transition probability heatmap at 3 years. (D) Predicted state transition probability heatmap at 4 years. The x-axis represents the initial CKM stage, and the y-axis represents the predicted CKM stage at each corresponding time horizon. Color intensity represents the magnitude of the predicted transition probability. CKM, cardiovascular-kidney-metabolic syndrome.

**Figure S2. Four-year observed transition proportions between low-risk and high-risk CKM states according to TyG tertiles.** (A) Four-year observed progression proportions from State 1 to State 2 according to TyG tertiles. (B) Four-year observed reversal proportions from State 2 to State 1 according to TyG tertiles. State 1, low-risk state defined as CKM stages 0-2; State 2, high-risk state defined as CKM stages 3-4; CKM, cardiovascular-kidney-metabolic syndrome; TyG, triglyceride-glucose index; T1, T2, and T3, first, second, and third tertiles of the TyG index, respectively.

**Figure S3. Dose-response relationship between TyG and the risk of transition from State 1 to State 2.** The figure shows the dose-response association between TyG and the risk of transition from State 1 to State 2 based on a restricted cubic spline model. The solid line represents the hazard ratio (HR), the shaded area represents the 95% confidence interval (CI), and the dashed horizontal line represents the reference line of HR = 1. The overall association was statistically significant (P for overall trend = 0.005), with no evidence of nonlinearity (P for nonlinearity = 0.500). State 1, low-risk state defined as CKM stages 0-2; State 2, high-risk state defined as CKM stages 3-4; TyG, triglyceride-glucose index; CKM, cardiovascular-kidney-metabolic syndrome; HR, hazard ratio; CI, confidence interval.

**Figure S4. Dose-response relationship between TyG and the risk of transition from State 2 to State 1.** The figure shows the dose-response association between TyG and the risk of transition from State 2 to State 1 based on a restricted cubic spline model. The solid line represents the hazard ratio (HR), the shaded area represents the 95% confidence interval (CI), and the dashed horizontal line represents the reference line of HR = 1. The overall association was not statistically significant (P for overall trend = 0.266), with no evidence of nonlinearity (P for nonlinearity = 0.220). State 1, low-risk state defined as CKM stages 0-2; State 2, high-risk state defined as CKM stages 3-4; TyG, triglyceride-glucose index; CKM, cardiovascular-kidney-metabolic syndrome; HR, hazard ratio; CI, confidence interval.
